# Migratory behaviour does not alter cophylogenetic congruence between avian hosts and their haemosporidian parasites

**DOI:** 10.1017/S0031182022000154

**Published:** 2022-06

**Authors:** Daniela de Angeli Dutra, Alan Fecchio, Érika Martins Braga, Robert Poulin

**Affiliations:** 1 Department of Zoology, University of Otago, Dunedin, New Zealand; 2 Programa de Pós-graduação em Ecologia e Conservação da Biodiversidade, Universidade Federal de Mato Grosso, Cuiabá, MT 78060-900, Brazil; 3 Departamento de Parasitologia, Instituto de Ciências Biológicas, Universidade Federal de Minas Gerais, Belo Horizonte, Brazil

**Keywords:** Avian haemosporidians, migratory behaviour, parasite–host cospeciation, phylogenetic congruence

## Abstract

Parasites display various degrees of host specificity, reflecting different coevolutionary histories with their hosts. Avian hosts follow multiple migration patterns representing short but also long distances. As parasites infecting migratory birds are subjected to multiple environmental and biotic changes through their flyways, migration may disrupt or strengthen cophylogenetic congruence between hosts and parasites. On the one hand, parasites might adapt to a single migratory host, evolving to cope with the specific challenges associated with the multiple habitats occupied by the host. On the other, as migrants can introduce parasites into new habitats, higher rates of host switching could also disrupt cophylogenetic patterns. We analysed whether migratory behaviour shapes avian haemosporidian parasite–host cophylogenetic congruence by testing if contributions of host–parasite links to overall congruence differ among resident and short-, variable- and long-distance migrants globally and within South America only. On both scales, we found significant overall cophylogenetic congruence by testing whether overall congruence differed between haemosporidian lineages and bird species. However, we found no difference in contribution towards congruence among links involving resident *vs* migratory hosts in both models. Thus, migratory behaviour neither weakens nor strengthens bird–haemosporidian cophylogenetic congruence, suggesting that other avian host traits are more influential in generating phylogenetic congruence in this host–parasite system.

## Graphical abstract

**Figure:**
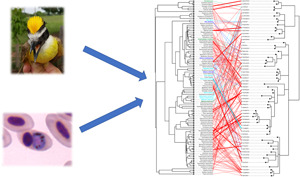

## Introduction

Parasites rely on their hosts to complete their life cycle and this strong dependence can favour high-evolutionary rates of cospeciation among parasites and their hosts (Poulin, 2007; Clayton *et al*., 2015). Cospeciation, i.e. the parallel speciation of hosts and parasites, can lead to the two sets of interacting organisms having similar phylogenies when each parasite species is aligned with its associated host (Clayton *et al*., 2015). However, mutual adaptation is not the only process driving cophylogenetic congruence among parasites and their hosts. Earlier research has identified other drivers of host–parasite phylogenetic congruence, such as dispersal and shared biogeographic histories (Park *et al*., 2020). Indeed, highly specialized parasites are more likely to present clear phylogenetic congruence with their hosts (Poulin, 2007). At the same time, Hay *et al*. (2018) showed that connectivity can also drive host–parasite coevolution by preventing differences in the genetic structure of parasites across host populations. Furthermore, parasites infecting hosts with high-dispersal ability may be less likely to cospeciate, and more likely to host switch (Ricklefs *et al*., 2004; De Vienne *et al*., 2013). Parasites of mobile hosts should be exposed to a wide range of other potential hosts and many opportunities for host switching; this would reduce any signal of cophylogenetic congruence as spillover events into new hosts disrupt congruence between parasite and host phylogenies (Ricklefs *et al*., 2004; Fecchio *et al*., 2018). Thus, the migratory behaviour of hosts should greatly influence the likelihood of host–parasite cospeciation and host switching by transporting parasites across different localities and facilitating the colonization of new host species (Poulin and de Angeli Dutra, 2021).

Migratory behaviour is a common feature across animals, being observed in many species of insects, turtles, fish, mammals and birds. Migratory birds represent around 19% of all avian species and can have profound effects on host–parasite relationships at the population and community levels (Bauer and Hoye, 2014). Earlier studies have revealed that migratory behaviour can both protect hosts against parasites or increase their risk of infection, with migratory species subject to either lower or higher parasite prevalence and richness, depending on the taxa under study (Koprivnikar and Leung, 2015; Teitelbaum *et al*., 2018; de Angeli Dutra *et al*., 2021*a*; Fecchio *et al*., 2021). These contrasting observations result from a balance of pressures between escaping parasites, increased exposure to infective stages and the physiological cost of migration (Poulin and de Angeli Dutra, 2021). Furthermore, parasite virulence favours the emergence of migration as an escape mechanism as higher virulence increases the benefit of escaping from pathogens (Balstad *et al*., 2021). The proportion of migrant individuals in a local bird assemblage may also alter local parasite prevalence and richness (de Angeli Dutra *et al*., 2021*b*; Fecchio *et al*., 2021). Indeed, migrant birds present greater centrality values within avian–haemosporidian networks (de Angeli Dutra *et al*., 2021*c*) and can drive the expansion of geographical range of haemosporidian parasites by introducing them into new regions and habitats (de Angeli Dutra *et al*., 2021*b*).

Avian haemosporidian parasites are cosmopolitan obligate vector-borne parasites that use haematophagous Diptera as their vectors and definitive hosts (i.e. host in which sexual exchange takes place) (Valkiūnas, 2005). Haemosporidians belonging to the genera *Plasmodium*, *Haemoproteus* and *Leucocytozoon* are highly diverse and prevalent in bird assemblages, having been intensely studied in ecological and evolutionary research worldwide (Marzal, 2012; Fecchio *et al*., 2020). As haemosporidian parasites infect migratory birds they face diverse environmental and biotic modifications as they are transported along their hosts' flyways, migratory behaviour could disrupt or favour phylogenetic congruence between hosts and their haemosporidian parasites. Haemosporidian parasites present variable levels of virulence towards their avian hosts and can be especially pathogenic to naïve species, such as penguins and endemic species from Hawaii and New Zealand that did not coevolve with the parasites (van Riper *et al*., 1982; Valkiūnas, 2005; Vanstreels *et al*., 2016). Indeed, host switching, migration and dispersal have already been linked to haemosporidian diversification and distribution in South America (Alcala *et al*., 2017; Fecchio *et al*., 2018; de Angeli Dutra *et al*., 2021*b*).

Here, we predict that host migration could either (1) increase the likelihood of a haemosporidian parasite shifting to new hosts through greater opportunities or (2) promote specialization of haemosporidian parasites for a subset of phylogenetically related migratory hosts following their adaptation to the particular conditions arising from the shifting fauna and abiotic conditions associated with host migration. Using a worldwide avian haemosporidian database (http://130.235.244.92/Malavi/; Bensch *et al*., 2009), we conducted Procrustean approach to cophylogeny (PACo) analyses to confirm whether haemosporidians and their avian hosts (mainly passerines) show evidence of cophylogenetic congruence, a pattern that would result mainly from frequent cospeciation and little host switching. Specifically, we also test whether haemosporidian lineages associated with migratory hosts contribute more or less to any cophylogenetic congruence pattern than haemosporidian parasites of non-migratory hosts, and/or whether the migratory distance covered by the host matters. As macroecological and evolutionary patterns can differ between distinct scales (Ricklefs, 2008), we then evaluate whether the association between migratory behaviour and phylogenetic congruence differs between global and regional (continental) scales.

## Materials and methods

### Dataset

**Fig. 1.**
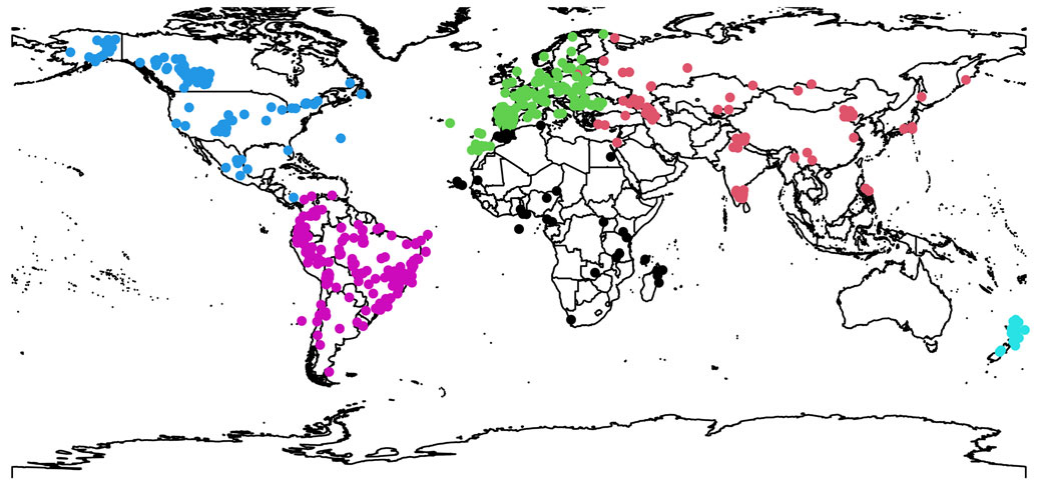
Distribution of sites where haemosporidian parasites have been recorded. A total of ~2200 from 430 sites (including offshore islands) were extracted from the MalAvi database. Colours represent distinct regions worldwide.

We extracted the dataset on haemosporidian lineages from the MalAvi database (http://130.235.244.92/Malavi/; Bensch *et al*., 2009) including all the data available from the ‘Grand Lineage Summary’ representing *Plasmodium* and *Haemoproteus* genera from wild birds, which also included information regarding locations where each lineage was recorded. The lineages were defined based on a cytochrome *b* fragment of 478 bp. Here, we considered only haemosporidian lineages and host species that were represented at least five times in the dataset, thus excluding rare and potentially accidental host–parasite associations. The final dataset contains a total of ~2200 haemosporidian parasite sequences representing 174 distinct lineages (101 *Plasmodium* and 73 *Haemoproteus*) collected from 347 different host species and representing 430 localities around the globe (Fig. 1). All bird species were classified as resident, short-distance migrant, variable-distance migrant or long-distance migrant according to Dufour *et al*. (2020). Then, in order to repeat the analyses also at a regional scale, we filtered the global dataset retaining only records from South America; we focused on that continent as it harbours the greatest diversity of avian hosts and haemosporidians worldwide (Jetz *et al*., 2012; Ellis *et al*., 2019). This regional dataset is composed of 482 haemosporidian sequences which comprise 59 distinct lineages (45 *Plasmodium* and 14 *Haemoproteus*), 101 host species and 113 localities.

### Parasite and host phylogenetic data

To infer the haemosporidian phylogeny from the sequence data, we ran a Bayesian phylogenetic reconstruction using MrBayes 3.2.2 (Ronquist and Huelsenbeck, 2003) with the GTR + I + G model of nucleotide evolution, as indicated by ModelTest (Posada and Crandall, 1998). We employed four Markov chains simultaneously for a total of 7.5 million generations that were sampled every 1000 generations. The first 1250 million trees were discarded as a burn-in step and the remaining trees were used to calculate the posterior probabilities of each estimated node in the final consensus tree. We considered *Leucocytozoon caulleryi* as the outgroup to root the phylogenetic tree.

Host phylogenetic trees were extracted from AllBirdsHackett1.tre from the https://birdtree.org/ website, which provides a global molecular phylogeny of birds. Then, we created a treeman file containing all trees from the original file using the ‘treeman’ package (Bennett *et al*., 2017) and randomly selected a consensus tree. Thereafter, all bird species from the consensus tree which were not present in the parasite dataset were removed.

### PACo analyses

To test whether haemosporidians and their avian hosts show significantly congruent phylogenies, and to calculate the contribution of each individual parasite–host link to overall congruence, we used the PACo package (Balbuena *et al*., 2013) implemented in R (Atkinson *et al.*, 2009). Firstly, we created two phylogenetic matrices representing hosts and parasites and phylogenetic distances between taxa, as well as a parasite–host interaction matrix. Host and parasite distance matrices were converted into principal coordinates matrices and taxa were duplicated to include multiple host–parasite interactions when necessary. Afterwards, we performed a goodness-of-fit test using 100 000 permutations to estimate whether parasite–host phylogenetic congruence was greater than expected by chance. In each permutation, we compared the sum of parasite–host congruence-squared values generated from our dataset to random values generated in each permutation. Afterwards, we calculated the contribution of each individual parasite–host link to the global fit by employing a jackknife procedure that estimates the squared residual of each individual link. A small residual value indicates a link that contributes to the overall congruence between host and parasite phylogenies, whereas a high residual value indicates the opposite. All analyses above were then repeated using the South American dataset. Parasite and host tanglegrams were plotted using the ‘cophylo’ function from the ‘phytools’ package in R (Revell, 2012) for South America only because the global dataset included too many taxa to allow comprehensible visualization of associations between hosts and their parasites.

### Bayesian analyses

We tested the effect of host migratory behaviour on the contribution of host–parasite links to overall cophylogenetic congruence with Bayesian models, implemented using the function ‘brm’ from the ‘brms’ package (Bürkner, 2017). Here, we considered cophylogenetic-squared residual values for each link as the response variable and used model averaging to estimate the best-fitted model. We employed the function ‘loo_model_weights’ applying both stacking and pseudo-BMA with Bayesian bootstrap methods. According to our preliminary analyses, the best-fitted model employed only the avian host migratory category (four levels: resident, short-distance migrant, variable-distance migrant and long-distance migrant; reference level = resident) as the population-level effect (Supplementary Table 1). Using the host phylogenetic tree, we created a matrix with phylogenetic distances between bird species. This matrix was also used in all the models as random effect to account for the host phylogenetic influence on the contribution of individual links to overall parasite–host cophylogenetic congruence. The host–parasite link ID was also added as random effect to account for the other characteristics of each link (e.g. sampled locality, number of interactions, etc.). Priors were determined using the ‘get_prior’ function. We ran the Bayesian models using four chains with 4000 total iterations per chain (2000 for warm-up, 2000 for sampling) and employed gamma distribution link equal to logarithm. Later, we reran the best-fitted Bayesian model for the South American dataset, and for *Plasmodium* and *Haemoproteus* parasites separately at both the global and regional scales.

## Results

**Fig. 2.**
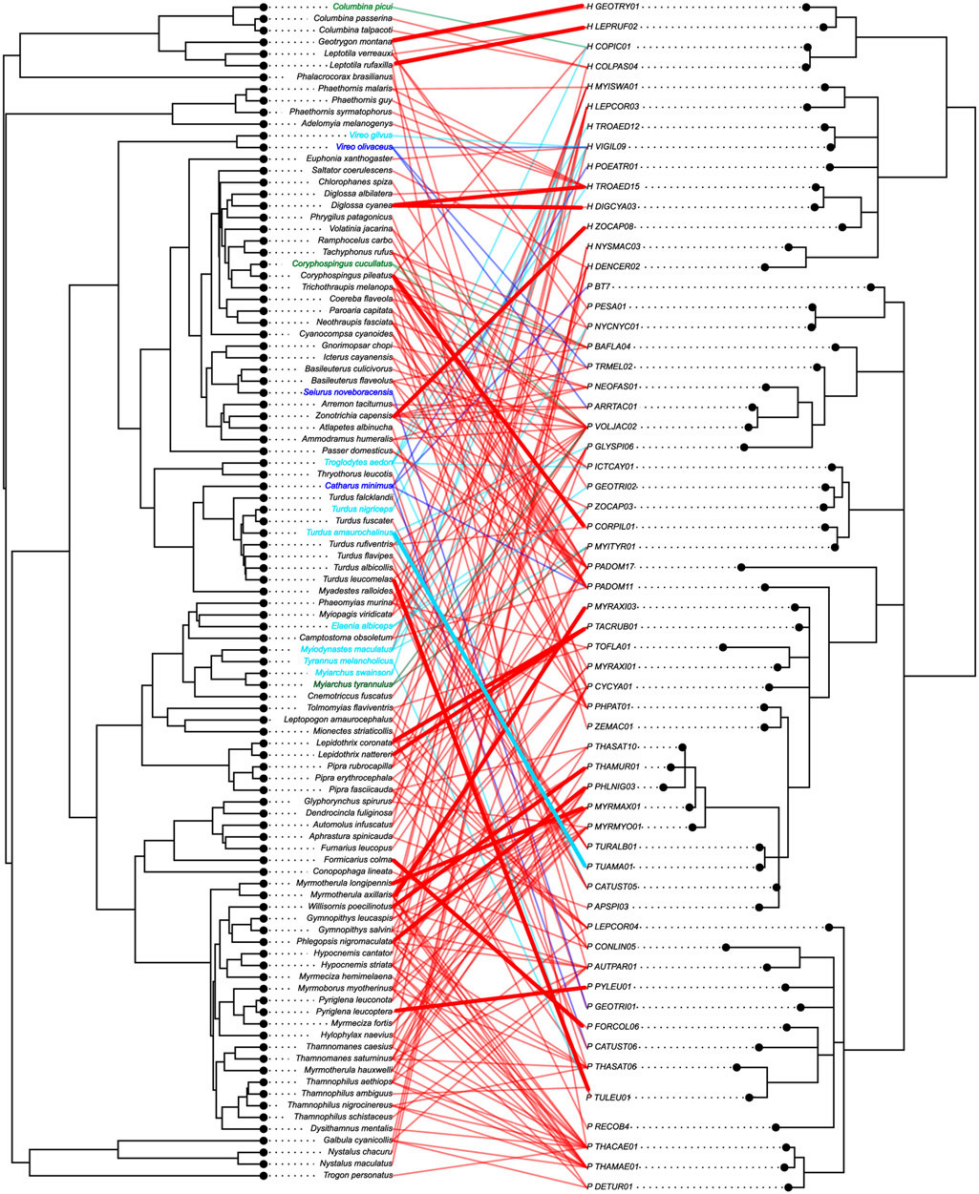
Tanglegram illustrating associations between haemosporidian parasites and their avian hosts from South America. Colours represent host species classified based on their distinct migratory distances: green, short distance; light blue, variable distance and dark blue, long distance. Black labels represent resident birds. Link density, i.e. the frequency of interactions as reported in the MalAvi database, is represented by line thickness: thin links, ≤5 interactions reported; thick links, ≥5.

Out of the 347 bird species in the global dataset, 209 were classified as residents while 33, 55 and 50 were considered short-, variable- and long-distance migrants, respectively. For South America, the dataset included 87 resident species and 3, 8 and 3 short-, variable- and long-distance migrants. Most birds were in the Passeriformes order in both datasets representing 94 and 89% of all individuals in the global and South American datasets. Similarly, birds from three continents (South and North America and Europe) each represented ~25% of our global dataset with most tropical species (~67%) sampled in South America. Haemosporidian parasites and their avian hosts showed phylogenetic congruence greater than expected by chance on both global and regional scales (PACo test, *P* < 0.00001 on both scales; see Fig. 2). Thus, we rejected the null hypothesis of random association among haemosporidians and their hosts and accepted the alternative hypothesis that avian haemosporidian evolutionary history is associated with that of their vertebrate hosts.

**Fig. 3.**
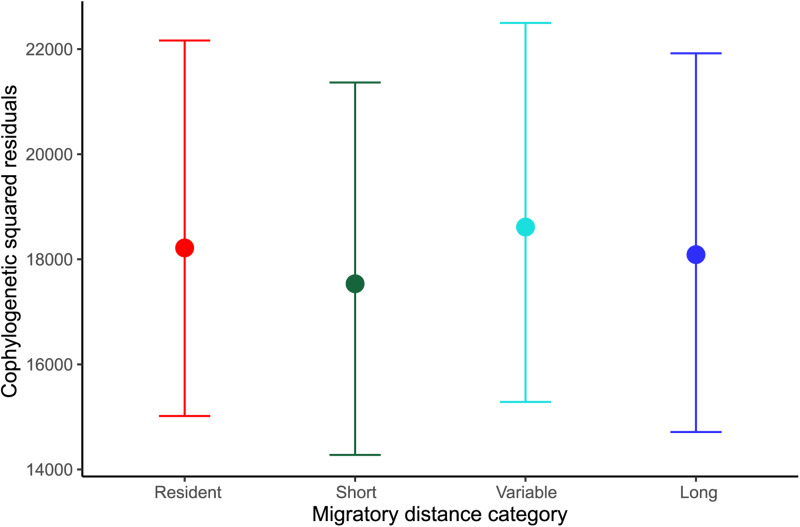
Mean (±credible intervals) squared residual of each individual parasite–host link according to the migratory category in which the bird hosts are classified in the global dataset.

**Fig. 4.**
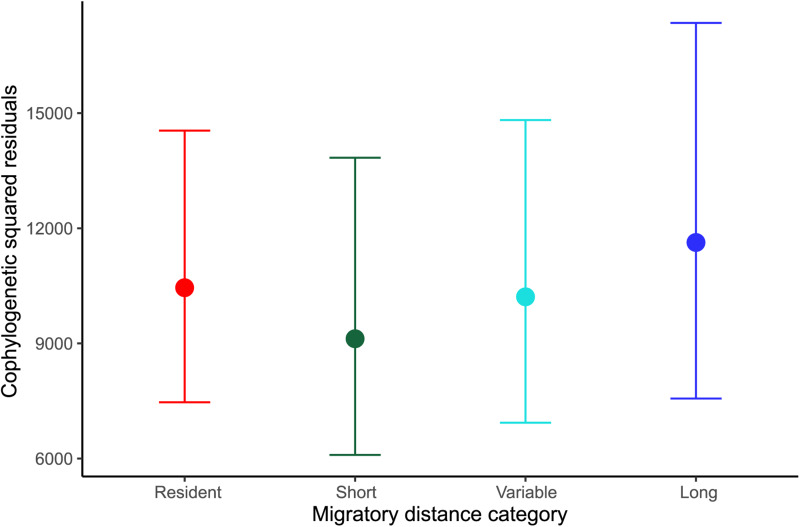
Mean (±credible intervals) squared residual of each individual parasite–host link according to the migratory category in which the bird hosts are classified in the South American dataset.

In the Bayesian models evaluating the effect of host migratory distance category on the squared residual values of each individual link (i.e. their contribution to overall cophylogenetic congruence), we found that hosts contribute equally to parasite–host cospeciation independently of whether or not they migrate, or their migration distance (Figs 3 and 4, Tables 1 and 2). We also found that both parasite–host association link ID and host phylogeny influenced the contribution of individual links to overall cophylogenetic congruence, as measured by the squared residuals. Additionally, the same patterns were observed in all models independently of scale (global or South America) or parasite taxa considered (*Plasmodium* only, *Haemoproteus* only or both genera combined; see Supplementary Tables 2–5).

**Table 1.** Parameter estimates, standard errors and confidence intervals for the Bayesian model testing the relationship between the cophylogenetic-squares residuals of each parasite–host link involving avian haemosporidian parasites and hosts of different migratory categories in the global dataset (reference level = resident)

	Estimate	Standard error	Confidence interval (95%)	
Intercept	9.82	0.09	9.63	9.99
Short distance	−0.04	0.03	−0.09	0.02
Variable distance	0.02	0.02	−0.03	0.07
Long distance	−0.01	0.03	−0.07	0.05
Host–parasite link ID	0.12	0.00	0.12	0.13
Host phylogeny	0.24	0.01	0.22	0.27

**Table 2.** Parameter estimates, standard errors and confidence intervals for the Bayesian model testing the relationship between the cophylogenetic-squared residuals of each parasite–host link involving avian haemosporidian parasites and hosts of different migratory categories in the South American dataset (reference level = resident)

	Estimate	Standard error	Confidence interval (95%)	
Intercept	9.25	0.17	8.92	9.58
Short distance	−0.13	0.13	−0.39	0.12
Variable distance	−0.01	0.09	−0.19	0.17
Long distance	0.12	0.09	−0.15	0.40
Host–parasite link ID	0.22	0.01	0.20	0.24
Host phylogeny	0.33	0.04	0.26	0.40

## Discussion

Parasite–host cospeciation occurs when both clades speciate in tandem, creating congruent phylogenies, which can occur due to neutral processes such as host dispersal and isolation or due to host-driven selection such as host defence (Clayton *et al*., 2003). Here, we demonstrate that haemosporidians and their avian hosts show cophylogenetic congruence on both regional and global scales, thus suggesting host diversification drove haemosporidian speciation. Furthermore, we investigated whether migratory habits could play a role in shaping the patterns of parasite–host cospeciation by either increasing opportunities for host switching or strengthening the association with a particular host taxa through selection for haemosporidian adaptations to changing environments. We found that parasite–host links generally made similar contributions to cophylogenetic congruence regardless of the host's migratory strategy. Therefore, migration does not seem to impact parasite–host coevolutionary history. On the contrary, host phylogeny may play an important role in shaping haemosporidian cospeciation with their hosts. Hence, selection originating from other aspects of host ecology might play a more important role compared to migratory habits in determining haemosporidian evolution.

Host defences may favour parasite cospeciation and lead to congruent phylogenies between parasites and their hosts, if they select for specific counter-adaptations that limit parasite success to the original host species (Clayton *et al*., 2003). Additionally, in the case of intracellular parasites, such as haemosporidians, molecular and protein divergence among multiple host species may prevent infection of novel hosts by blocking cell invasion by the parasite (Horta *et al*., 2020). As expected, haemosporidians perform better within a subset of phylogenetically related hosts (Pinheiro *et al*., 2016) while the same haemosporidian lineage must express distinct genes when infecting different host species (Garcia-Longoria *et al*., 2020). However, even though we found evidence of cospeciation between haemosporidians and their hosts, earlier research has revealed that host shifts play an important role in avian haemosporidian diversification and can even disrupt their cophylogenetic congruence with hosts (Ricklefs *et al*., 2004; Alcala *et al*., 2017). Spillover events can also be more common for parasites transmitted by generalist vectors, which is the case for haemosporidians (Santiago-Alarcon *et al*., 2012; Martínez-de la Puente *et al*., 2015). Indeed, Ferreira-Junior *et al*. (2018) recently reported that *Plasmodium juxtanucleare*, a haemosporidian parasite previously observed only in Galliformes, infected wild passerines in Brazil. Such events can disrupt cophylogenetic congruence, leading to apparent incongruence between haemosporidians and their hosts (Ricklefs *et al*., 2004).

Furthermore, we demonstrated that host migratory behaviour does not affect haemosporidian cophylogenetic congruence, suggesting that other host features can be more important in determining haemosporidian infection of novel hosts, such as cell invasion ability or immunological defence. Similarly, even though migrants can disperse haemosporidians (de Angeli Dutra *et al*., 2021*b*), these migratory species may contribute only to parasite dispersal if host species in their new community are phylogenetically similar to their original migratory host. In addition, haemosporidians do not necessarily reduce avian migration success, or do so negligibly (Hahn *et al*., 2018; Kelly *et al*., 2018; Ágh *et al*., 2019). When they do, only birds presenting high parasitaemia or in the acute phase of infection might be unable to complete their migration (Altizer *et al*., 2011; Poulin and de Angeli Dutra, 2021). Indeed, dispersal has been proposed as one of the drivers of haemosporidian diversification in South America (Fecchio *et al*., 2018). Thus, haemosporidian-infected birds are often capable of completing their migration and contribute to parasite dispersal and, consequently, diversification. It is also important to note that haemosporidian diversification followed the radiation of modern avian fauna after the Cretaceous–Palaeogene boundary (Pacheco *et al*., 2018), thus, cophylogenetic congruence of haemosporidians and their hosts reinforces that parasite speciation and diversification were driven by bird evolution.

Vectors can also play a fundamental role in determining haemosporidian parasite transmission in nature (Yan *et al*., 2021). Blood-feeding behaviour of vectors may restrict haemosporidian transmission to the subsets of bird species due to the innate preferences mosquitoes display when choosing their targets (Yan *et al*., 2021). Vector specificity or innate preferences can thus contribute to restricting parasite transmission to ecologically similar bird species. Moreover, molecular/protein mismatch between parasites and vectors could also constrain parasite development to only certain phylogenetically related vectors with similar host preferences, thus further promoting parasite–host cophylogenetic congruence. The introduction of vectors to new regions can, however, allow generalist haemosporidians to increase their geographical and host range by expanding their opportunities for host switching. For instance, the introduction of *Culex quinquefasciatus* in Hawaii and New Zealand enabled the infection of local endemic and naïve fauna by *Plasmodium* species, especially the highly generalist *Plasmodium relictum* (Warner RE, 1968). In addition, the fact migrants are capable of dispersing haemosporidian lineages indicates that competent vectors are found throughout the birds' flyways (de Angeli Dutra *et al*., 2021*b*). Nevertheless, information regarding haemosporidian vector specificity and vector preferences is too limited at present to test these possibilities.

It is important to note, however, that Jenkins *et al*. (2012) identified avian migration, feeding behaviour, body size and nesting habitat as predictors of parasite–host coevolution for *Leucocytozoon*, another related genus of avian haemosporidian parasites. As we also observed, host phylogeny influenced parasite–host cophylogeny in all models, indicating that other phylogenetically conserved host traits may contribute towards parasite–host cophylogeny. However, the narrower climatic niche and higher host specificity of *Leucocytozoon* spp. (Valkiūnas, 2005; Reeves *et al*., 2015; Cuevas *et al*., 2020) may prevent parasites from shifting to new bird hosts, constraining parasite evolutionary history to their local resident hosts. Additionally, as highly specialized haemosporidians also tend to be more geographically restricted (de Angeli Dutra *et al*., 2021*d*), resident hosts might only be infected by lineages from the regional pool of *Leucocytozoon* parasites, whereas migrants may be exposed to parasites from multiple regions and continents. This pattern could also lead to links involving associations between *Leucocytozoon* lineages and resident hosts contributing more towards cophylogenetic congruence compared to those involving migrant hosts. It is worth mentioning, however, that our relatively small number of migrants, especially for the South American analyses, might have hidden an association between migratory status and haemosporidian–bird cospeciation.

Here, we demonstrate that haemosporidian parasites and their avian hosts generally share a common evolutionary history and show significantly congruent phylogenies. Furthermore, we also found that bird migration is not weakening or strengthening the congruence observed between parasites and their hosts, even when considering different spatial scales or the genera *Plasmodium* and *Haemoproteus* separately. Nonetheless, we detected an effect of host phylogeny on host–parasite cophylogenetic congruence, suggesting that congruence among parasites and their hosts may be linked to other host traits, and possibly also to vector specificity. It must be noted that, despite the fact haemosporidians and their avian hosts appear to have coevolved, these parasites are still capable of switching to phylogenetically distant hosts (Ferreira-Junior *et al*., 2018). Therefore, the influence of host phylogeny we observed suggests that haemosporidian coevolution with birds may be largely driven by host-imposed selection.

## Data Availability

All data that support the findings of this study is openly available at http://130.235.244.92/Malavi/ (Bensch *et al*., 2009).

## Supplementary material

For supplementary material accompanying this paper visit https://doi.org/10.1017/S0031182022000154. click here to view supplementary material
