# Supplementary material for: Migratory behaviour does not alter cophylogenetic congruence between avian hosts and their haemosporidian parasites
Source: Parasitology. 2022 Mar 7;149(7):905–12. doi: 10.1017/S0031182022000154 (PMC10090587; doi:10.1017/S0031182022000154)
Supplement: Supplementary file 1 [file S0031182022000154sup001.docx]

**Migratory behavior does not alter cophylogenetic congruence between avian hosts and their haemosporidian parasites**

Daniela de Angeli Dutra¹, Alan Fecchio², Érika Martins Braga³, Robert Poulin¹

Supplementary Table 1: Model averaging weights for the four models tested using stacking and pseudo-BMA (where higher values indicate better model fit) with Bayesian bootstrap method. Note: migration strategy has only three levels, resident, partial, strict and migratory (all distances considered together).

| Models | Stacking | Pseudo-BMA |
| --- | --- | --- |
| cophylogenetic square residuals ~ migration strategy | 0.260 | 0.012 |
| cophylogenetic square residuals ~ migration strategy+ migration distance in kilometers | 0.000 | 0.000 |
| cophylogenetic square residuals ~ migration distance in kilometers | 0.000 | 0.000 |
| cophylogenetic square residuals ~ migration distance in categories | 0.740 | 0.988 |

Supplementary Table 2: Parameter estimates, standard errors, and confidence intervals for the Bayesian model testing the relationship between the cophylogenetic square residuals of each parasite-host link involving *Plasmodium* parasites and hosts of different migratory categories in the global dataset (reference level = resident).

|  | Estimate | Stand. Error | Conf. Inter (95%) | |
| --- | --- | --- | --- | --- |
| Intercept | 9.75 | 0.10 | 9.55 | 9.96 |
| Short distance | -0.02 | 0.03 | -0.09 | 0.04 |
| Variable distance | 0.02 | 0.02 | -0.02 | 0.07 |
| Long distance | -0.01 | 0.03 | -0.06 | 0.05 |
| Host-parasite link ID | 0.07 | 0.02 | 0.01 | 0.11 |
| Host phylogeny | 0.25 | 0.01 | 0.22 | 0.27 |

Supplementary Table 3: Parameter estimates, standard errors, and confidence intervals for the Bayesian model testing the relationship between the cophylogenetic square residuals of each parasite-host link involving *Haemoproteus* parasites and hosts of different migratory categories in the global dataset (reference level = resident).

|  | Estimate | Stand. Error | Conf. Inter (95%) | |
| --- | --- | --- | --- | --- |
| Intercept | 9.76 | 0.12 | 9.52 | 9.99 |
| Short distance | -0.05 | 0.04 | -0.13 | 0.03 |
| Variable distance | 0.01 | 0.04 | -0.07 | 0.09 |
| Long distance | 0.01 | 0.04 | -0.07 | 0.10 |
| Host-parasite link ID | 0.08 | 0.03 | 0.01 | 0.12 |
| Host phylogeny | 0.29 | 0.02 | 0.26 | 0.33 |

Supplementary Table 4: Parameter estimates, standard errors, and confidence intervals for the Bayesian model testing the relationship between the cophylogenetic square residuals of each parasite-host link involving *Plasmodium* parasites and hosts of different migratory categories in the South American dataset (reference level = resident).

|  | Estimate | Stand. Error | Conf. Inter (95%) | |
| --- | --- | --- | --- | --- |
| Intercept | 9.23 | 0.18 | 8.86 | 9.58 |
| Short distance | -0.14 | 0.15 | -0.44 | 0.16 |
| Variable distance | -0.02 | 0.10 | -0.22 | 0.18 |
| Long distance | 0.14 | 0.15 | -0.15 | 0.44 |
| Host-parasite link ID | 0.19 | 0.03 | 0.12 | 0.23 |
| Host phylogeny | 0.33 | 0.04 | 0.26 | 0.42 |

Supplementary Table 5: Parameter estimates, standard errors, and confidence intervals for the Bayesian model testing the relationship between the cophylogenetic square residuals of each parasite-host link involving *Haemoproteus* parasites and hosts of different migratory categories in the South American dataset (reference level = resident).

|  | Estimate | Stand. Error | Conf. Inter (95%) | |
| --- | --- | --- | --- | --- |
| Intercept | 9.19 | 0.21 | 8.79 | 9.61 |
| Short distance | -0.07 | 0.34 | -0.71 | 0.61 |
| Variable distance | -0.06 | 0.19 | -0.43 | 0.30 |
| Long distance | -0.07 | 0.23 | -0.54 | 0.39 |
| Host-parasite link ID | 0.18 | 0.07 | 0.02 | 0.28 |
| Host phylogeny | 0.39 | 0.06 | 0.28 | 0.53 |
